# Individuality in Visual Representation of Pulse: A Comprehensive Analysis

**DOI:** 10.3390/s26196224

**Published:** 2026-09-30

**Authors:** Lu Sun, Liting Wang, Changsong Liu, Fengshan Bai, Zhiyi Ma

**Affiliations:** 1 Department of Mathematical Sciences, Tsinghua University, Beijing 100084, China; 2 Department of Electronic Engineering, Tsinghua University, Beijing 100084, China; 3 School of Clinical Medicine, Tsinghua University, Beijing 100084, China

**Keywords:** individuality, photoplethysmography (PPG), wearable, personalized health, phase–amplitude coupling

## Abstract

Individuality, a foundational concept of traditional medicine, has been increasingly challenged by the standardization of modern healthcare. The emergence of wearable technology offers a scalable pathway toward precision health. Here, we explored the comprehensive visual representation of wearable photoplethysmography signals from a single device using the phase–amplitude coupling method. We demonstrated that the coupling diagrams contain visually distinguishable regions of physiological frequency components, including those consistent with reference breathing rates (R-squared value of 0.86 for linear regression and mean absolute error of 0.017 Hz, i.e., 1.02 breaths/min) and related to reference heart rates (R-squared value of 0.89). A similarity analysis at 0.1 Hz revealed a complex relationship between photoplethysmography signals and the derived skin sympathetic nerve activity and pulse rate variability. The numerical coupling matrices were further utilized to achieve cross-day and cross-session individual identification through machine-learning classifiers. The two-dimensional CNN classifier performed the best in the cross-session scenarios, achieving 90.37% accuracy with an equal error rate of 2.86%. This comprehensive analysis depicts the overall physiological information and individuality contained in digital pulse signals, providing conceptual and methodological foundations for future personalized health research.

## 1. Introduction

The concept of individuality in health models, emphasized by Hippocrates in ancient Greece [1,2], resonates deeply with the modern vision of precision medicine [3,4]. For decades, standardized approaches have dominated the healthcare system [2,5], driving the development of uniform care protocols. Yet these one-size-fits-all strategies are well-known to be insensitive to individual variability [2,5]. A substantial fraction of patients derive limited benefit from drugs in population-based clinical trials, and such trials may even increase the risk of adverse drug events [6,7]. Against the backdrop of standardized measurement [8] and large-scale manufacturing, there are both opportunities and challenges to realizing the full potential of precision medicine [4,5,7,9,10,11].

The digital health revolution driven by wearable technology [11,12] is central to achieving population health beyond hospitals [4,9,13,14] and promoting health equity at scale [15]. Passively collected, longitudinal, and routinely tracked health information have enabled predictions of several clinical laboratory measurements [14], disease identification [13,16], and early detection of adverse physiological events [11,17] at the individual level. However, as technology advances and nosology becomes increasingly refined [4], countless indicators and biomarkers have been proposed [18]. This brings immediate challenges in information explosion [4], data fusion [19], and multimodal sensor development [18]. Moreover, the assessment of overall health requires a multi-dimensional understanding [10], whereas isolated data points provide only partial descriptions of specific conditions [19].

We explored the comprehensive physiological representation of wearable data from a single photoplethysmography (PPG) device, rather than integrating individually obtained indicators. PPG signals capture subtle blood volume fluctuations that reflect pulse wave changes and contain rich frequency information related to hemodynamics [20], respiration [21,22], and autonomic nervous activity [23,24]. Conventional PPG analysis extracting indicators in isolation can only obtain limited information about complex health issues.

Converting one-dimensional PPG signals into graphic forms facilitates intuitive observation and subsequent analysis about health information, such as the effective use of neural networks [25]. The visibility graph [26] and spectrogram [27] are common graphical representations. They express the dynamic information of original PPG signals, but provide limited visual interpretability and discard phase information. This study introduced the phase–amplitude coupling (PAC) method to PPG analysis, which was originally developed for electroencephalography and magnetencephalography [28,29,30]. PAC captures interactions between the phase of a low-frequency oscillation and the amplitude of a high-frequency oscillation. In recent years, PAC has extended beyond the brain, such as into gastric rhythm [31], respiration [32], and heart rate variability (HRV) [33]. However, most of these studies only focus on limited specific indicators and lack comprehensive analysis of health information.

**Figure 1:**
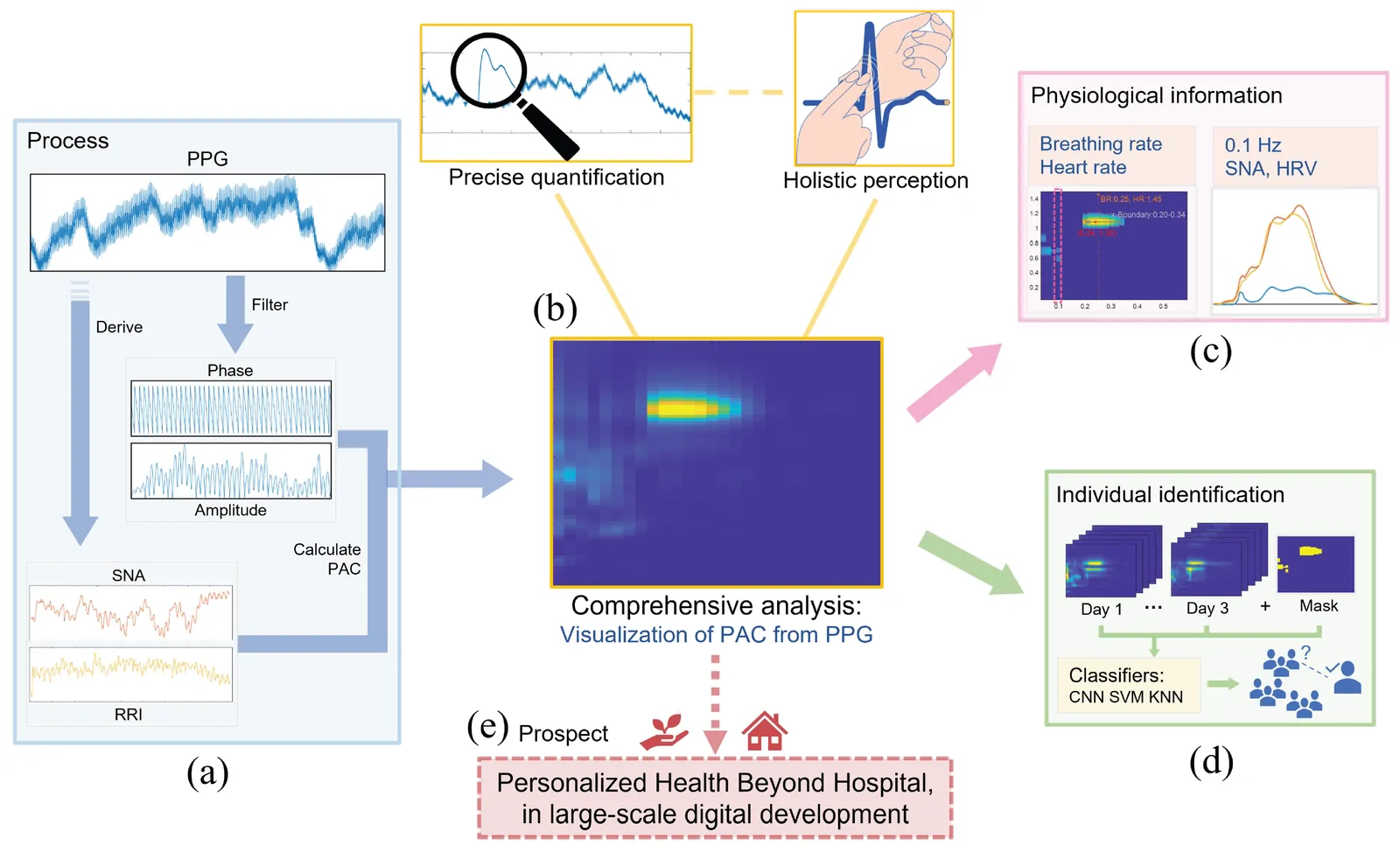
A scheme for the visual representation of pulse. (**a**) The modulation index matrices and pseudo-color diagrams were generated from photoplethysmography (PPG) signals as well as the derived sympathetic activity (SNA) and RR interval signals (RRI) using the phase–amplitude coupling method (PAC). (**b**) PAC visualization from PPG signals provides a comprehensive representation of quantitative analysis and holistic visual inspection. (**c**) The PAC diagrams contained physiological information, including breathing rate, heart rate, and 0.1 Hz fluctuations associated with sympathetic activity and heart rate variability. (**d**) Machine-learning classifiers achieved individual identification in the cross-day and cross-session scenarios. (**e**) Comprehensive analysis of PPG could advance personalized health monitoring beyond the hospital, alongside facilitating the development of digital technology.

In this study, we comprehensively translated the visual representation of PPG signals and conveyed the individuality of pulse with physiological interpretation (Figure 1). The PAC method was introduced for graphical representation of the pulse (Figure 1b). The physiological components in PAC diagrams were quantitatively analyzed (Figure 1c), including the respiratory rate, heart rate, and 0.1 Hz fluctuations. We further achieved cross-day and cross-session individual identification using the modulation index (MI) matrices through machine-learning algorithms, focusing on the frequency-domain perspective (Figure 1d). The integration of wearable technology with comprehensive analysis enables both physiological profiling and individual identification, potentially advancing personalized digital health monitoring.

## 2. Materials and Methods

### 2.1. PPG Datasets

Three finger PPG datasets were selected for different experimental purposes. Firstly, we conducted a preliminary parameter-sensitivity analysis on 8 min PPG segments of spontaneous breathing from the CapnoBase dataset [34]. The parameters determined were used for subsequent PAC experiments.

The MARSH dataset [35] was introduced for visual representation and quantitative analysis of physiological frequency components. It contained impedance pneumography with annotations and raw finger PPG signals from 29 healthy subjects (mean age 24.17 ± 4.19 years, 5 females) in sitting posture at a sampling rate of 250 Hz. The first 3 min eupnea segment from each subject was selected, along with the reference respiration rate from impedance pneumography and heart rate from the electrocardiogram. Both reference signals were recorded simultaneously with the PPG signals.

The third dataset was self-collected for individual identification. It consisted of PPG signals from both the left and right index fingers of 20 adults (mean age 36.3 ± 8.95 years, 10 females) in sitting posture. Each PPG segment was 3 min long in transmission mode at a 500 Hz sampling rate. PPG signals were acquired over three consecutive days, with at least 30 min between recording sessions and at least five records per day per subject. The dataset contained a total of 644 segments. All recordings were performed in a controlled laboratory, with a 5 min resting period before each session. The same PPG device (OEM-S3951A, Med-linket Medical Electronics Co., Ltd., Shenzhen, China) and 660–905 nm red and infrared wavelength probe were used for all subjects. None of the participants had cardiovascular, respiratory, or neurological diseases. All experimental protocols were approved by Beijing Tsinghua Changgung Hospital Ethic Committee (approval number 24556-0-01). Informed consent was obtained from all subjects. All experiments were performed in accordance with relevant guidelines and regulations.

### 2.2. Phase–Amplitude Coupling of PPG

Coupling diagrams and modulation index matrices were generated based on the PAC algorithm of Tort et al. [28]. In Figure 1a, the raw PPG signals were filtered with complex Morlet wavelets [29] to obtain instantaneous phase ϕ(t) and amplitude A(t) sequences at specific frequency bands. The phase angle [0,2π) was divided into $N_{\mathrm{bins}}$ bins, and the average amplitude ${\langle A\rangle }_{j}$ within each bin *j* was calculated. The Kullback–Leibler distance between the normalized amplitude distribution $P\left(j\right)={\langle A\rangle }_{j}/\sum_{k}{\langle A\rangle }_{k}$ and a uniform distribution $U\left(j\right)=1/N_{\mathrm{bins}}$ was: $D_{\mathrm{KL}}\left(P∥U\right)=\sum_{j=1}^{N_{\mathrm{bins}}}P\left(j\right)\log\left[\frac{P(j)}{U(j)}\right]$. The MI value was normalized as: $\mathrm{MI}\left(f_{\mathrm{phase}},f_{\mathrm{amp}}\right)=\frac{D_{\mathrm{KL}}\left(P∥U\right)}{\log(N_{\mathrm{bins}})}$. The modulation indices at two frequency bands formed the MI matrix, from which pseudo-color coupling diagrams were derived.

In this paper, the frequency ranges for phase and amplitude sequences were 0.04–0.6 Hz and 0.04–1.5 Hz, with a 0.02 Hz step. The upper limit frequency of 1.5 Hz covers all heart rates in MARSH and self-collected datasets; for higher heart rates this limit should be increased. The low-band range included 0.1 Hz and the general respiratory rate. Parameters (wavelet width 7, $N_{\mathrm{bins}}$ 20) were selected based on the PAC literature [28,29,30] and PPG frequency content [20,21,22,23,24], which were analyzed in the parameter-sensitivity experiments.

All calculations were implemented with MATLAB 2023a. Because the wavelet transform inherently performed bandpass filtering at each frequency band pair, no additional preprocessing was applied on PPG signals, including normalization or motion-artifact removal. However, for raw PPG signals that retained the envelope trend, first-order differencing (i.e., subtraction of adjacent sampling points) was applied to obtain differential PPG (DPPG), which improved visual distinguishability. For already de-trended PPG signals (e.g., the CapnoBase dataset), no difference operation was needed.

The computational complexity of the PAC method can be estimated from its algorithmic structure. For a PPG segment of N sample points and a frequency band pair, the time complexity of complex Morlet wavelet convolution is O(NlogN), while that of phase extraction and binning is O(N) and mean amplitude per bin is $O(N_{\mathrm{bins}})$. The total complexity is $O(F_{p}\times F_{a}\times N\log N)$ for $F_{p}$ phase frequencies and $F_{a}$ amplitude frequencies.

### 2.3. Parameter Sensitivity with the CapnoBase Dataset

**Figure 2:**
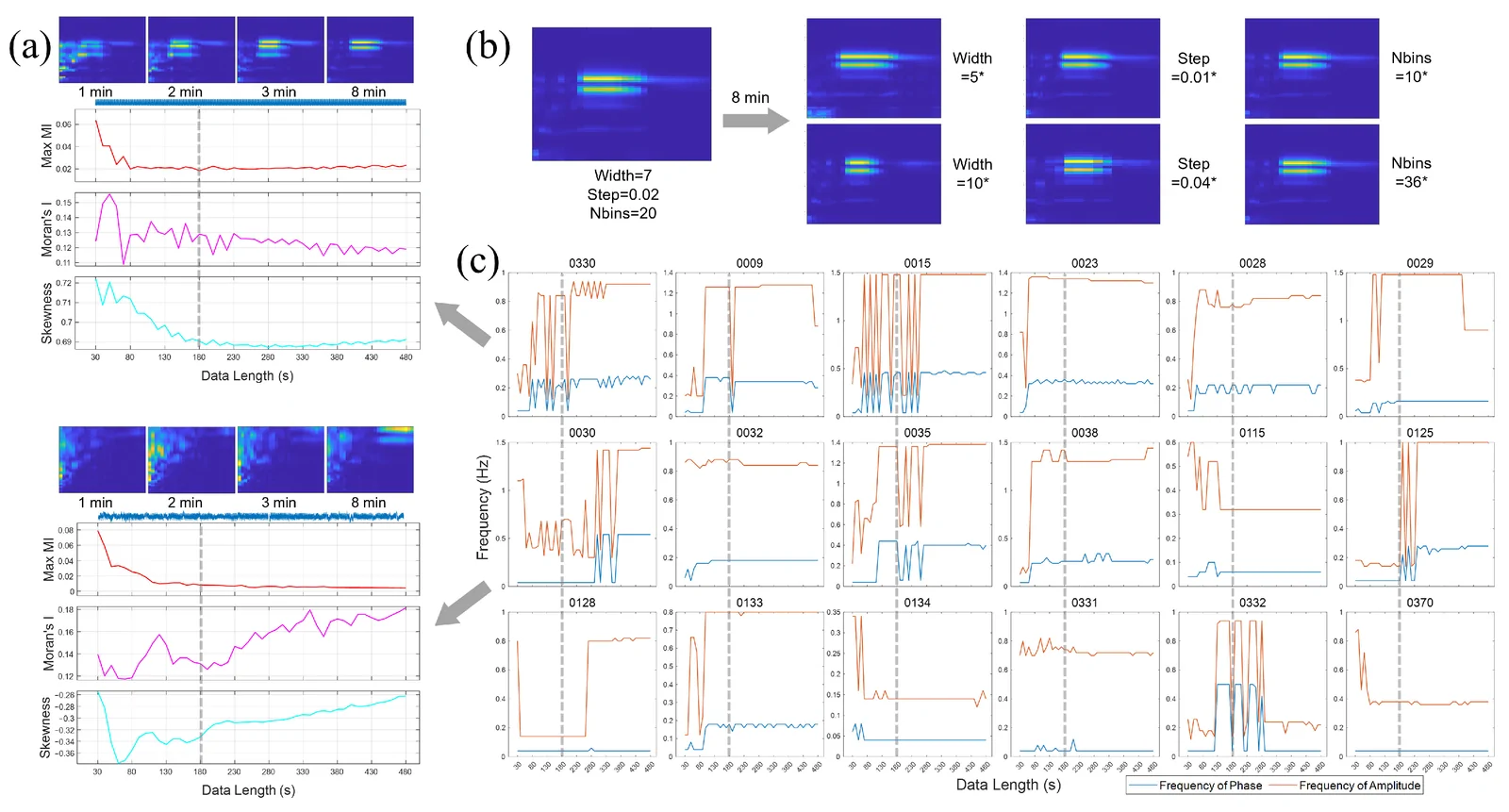
Parameter sensitivity analysis of the PAC algorithm based on the CapnoBase dataset. (**a**) Two examples of PPG signals’ skewness, maximum MI values (MAX MI), global Moran indices (Moran’s I), and PAC coupling diagrams across different data lengths. (**b**) Visual inspection of coupling diagrams under different parameters with 8 min PPG segments. Asterisks mark parameters changed relative to default settings. (**c**) Phase and amplitude frequencies at the maximum value position at different data lengths for 18 subjects. Gray dashed lines mark 180 s (3 min) of data length.

We conducted a sensitivity analysis of PAC parameters using 8 min spontaneous-breathing PPG segments from the CapnoBase dataset [34]. Because high-intensity coupling clusters may be related to individual heart rate and respiratory rate, it is inappropriate to analyze on fixed frequency bands. Therefore, we used specialized indicators to describe the coupling relationship within MI matrices and its dependence on data length (Figure 2). The maximum MI values (MAX MI) and corresponding phase and amplitude frequencies were recorded; the latter curves are presented in Figure 2c. The global Moran index (Moran’s I) [36] was introduced to evaluate the clustering effect of modulation indices, where positive values indicate clustering and zero values indicate spatial randomness. In addition, we adopted skewness [37,38] (‘skewness’ function in MATLAB) as a PPG signal-quality index at different data lengths, taking the average skewness of 5 s segments as the window. Figure 2a shows two examples of MAX MI values, Moran indices, and skewness values across data lengths from subjects ‘0330’ and ‘0030’. With sufficiently long fixed 8 min PPG segments, the coupling level under different parameters was inferred by visual inspection, shown in Figure 2b, as suggested by Tort et al. [28].

The maximum value and corresponding frequencies tended to stabilize as data length increased, typically reaching stability at 3 min (marked by gray dashed line in Figure 2c) for higher-quality signals. Figure 2a shows the results of two PPG signal qualities, with smaller negative skewness values indicating lower quality [37,38]. The global Moran indices of these MI matrices were positive, indicating the presence of coupling clustering but no stable relationship with data length. Through visual inspection in Figure 2b, the wavelet width parameter affected clustering size, while the highest-intensity position remained unchanged. The number of bins had no significant visual effect for PPG data, although more bins increased computation time. Smaller frequency steps yielded a finer coupling resolution at the cost of computation time.

On the other hand, the HRV analysis takes less than 5 min for the ultra-short term [39], supporting the feasibility of calculating HRV for 3 min segments. There are approximately 20 complete oscillations at around 0.1 Hz in a 3 min stable PPG segment, ensuring sufficient low-frequency components.

Therefore, taking into account the parameter selection in the literature, the trade-off between computational efficiency and resolution, and visual inspection, we performed PAC calculations on 3 min segments of high-quality PPG signals with default parameters. For other data types or research purposes, additional analyses of parameters and data length are still required [28].

### 2.4. Extraction and Analysis of Physiological Information with the MARSH Dataset

#### 2.4.1. Information Extraction from Regions in PAC Diagrams Related to Breathing Rate and Heart Rate with the MARSH Dataset

**Figure 3:**
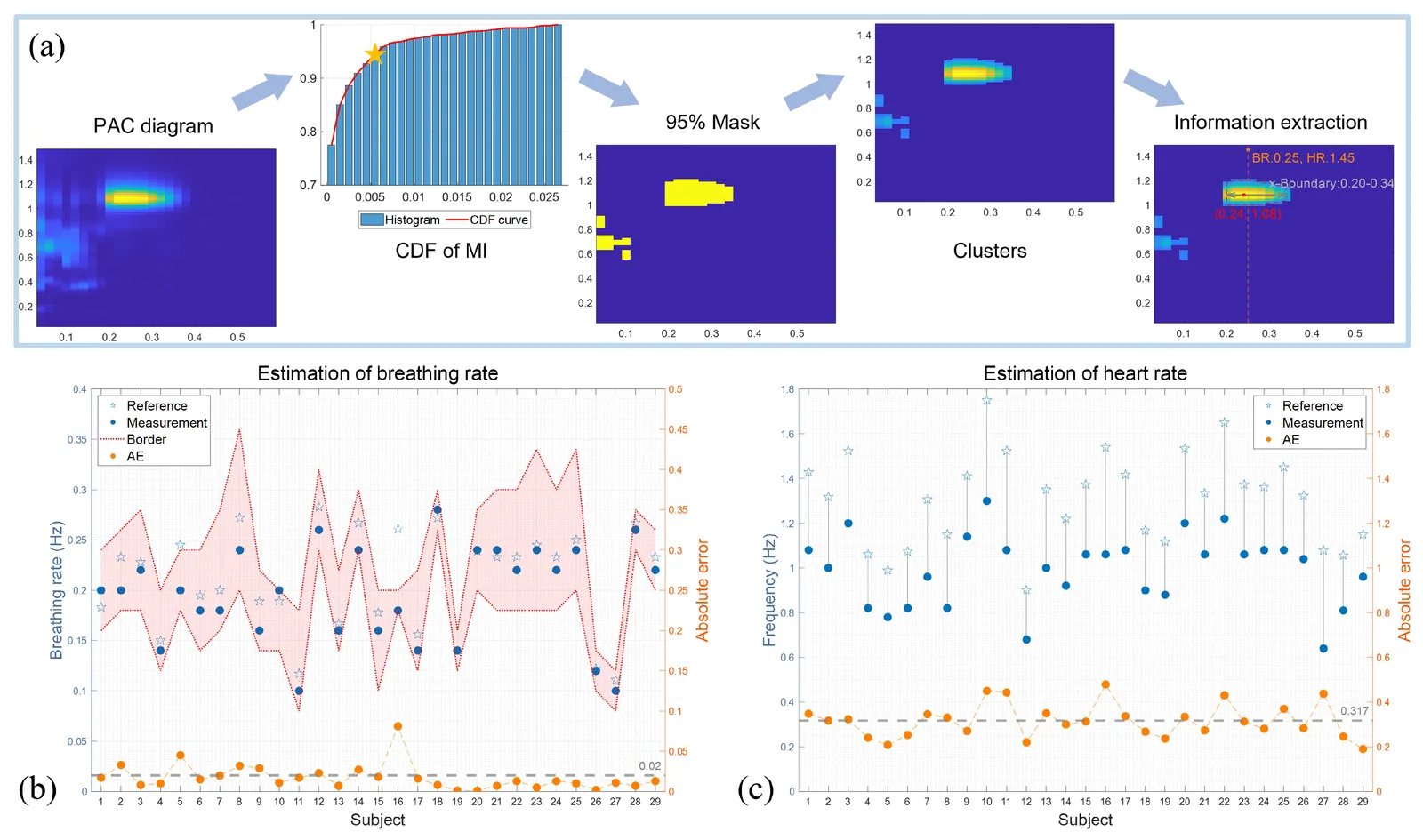
PAC diagrams contain frequency information about breathing and heart rate. (**a**) The cumulative distribution function (CDF) of modulation indices has a turning point near 0.95 (yellow star). Thus the top 5% of elements by intensity are retained to form a binary mask. Significant connected clusters are then identified. Coordinates of maximum-intensity points in each cluster are compared with the reference respiration rate and heart rate. (**b**) Extracted breathing rates (solid blue dots) are compared with reference values (blue hollow stars) for 29 people from the MARSH dataset. The red bands indicate the horizontal range of each cluster; orange dots below show absolute errors. (**c**) Ordinate values (solid blue dots) are compared with reference heart rates (blue hollow stars). Orange dots below show absolute errors.

We extracted significant regions from the PAC pseudo-color diagrams and compared the corresponding frequencies with reference respiration rates and heart rates, based on the MARSH dataset (Figure 3a).

The histogram distribution of MI matrix element values was not typically normal. The turning point of the cumulative distribution function (CDF) was approximately 0.95 on the vertical axis (yellow star in Figure 3a), indicating that the top 5% of values formed a distinct high-intensity region. Therefore, we generated a binary matrix by retaining the top 5% of elements by intensity (95% mask) and setting the remainder to zero. Significant connected clusters were then identified using the ‘bwlabel’ function (conn = 8). Within each cluster, the horizontal and vertical coordinates of maximum-intensity points were compared with the reference respiration rate and heart rate, respectively. The horizontal range of the significant region was defined by the first left and right intersections of a horizontal line from the maximum-intensity point with the cluster boundary.

#### 2.4.2. Analysis of 0.1 Hz Columns in PAC Diagrams

**Figure 4:**
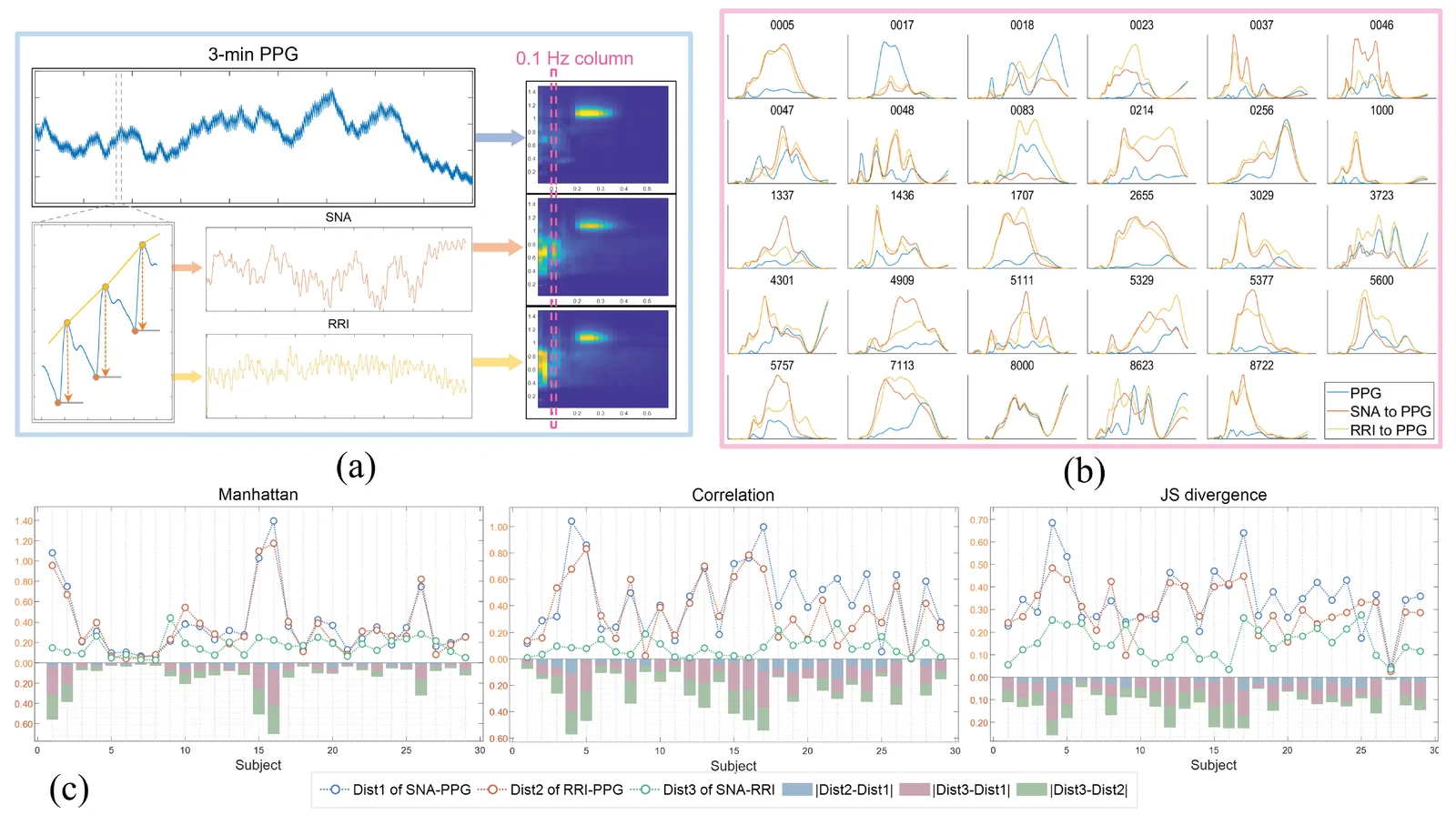
Separation of 0.1 Hz fluctuations in PPG signals using derived sympathetic activity (SNA) and heart rate variability in the PAC framework. (**a**) SNA and R-R interval (RRI) signals were derived from PPG signals, based on the peak location and peak-to-trough amplitude of each pulse. Phase–amplitude coupling diagrams of PPG self-coupling, SNA-to-PPG coupling, and RRI-to-PPG coupling are shown in the right panel. (**b**) Numerical distributions of the 0.1 Hz column from these three diagrams are presented for 29 subjects from the MARSH dataset. (**c**) Three similarity measures (Manhattan distance, correlation distance, Jensen–Shannon divergence) are used to compare the three 0.1 Hz distribution curves across subjects. The bar charts below show differences between distance pairs.

Another significant region at 0.1 Hz (corresponding to a 10 s oscillation period) was observed in the PAC diagram. The 0.1 Hz component of PPG has received persistent attention, which is associated with autonomic nervous activity [20]. We conducted an analysis of 0.1 Hz fluctuations using the MARSH dataset. Skin sympathetic nerve activity and pulse rate variability are the two main contributions to 0.1 Hz fluctuations in PPG signals, with the latter considered an effective surrogate for HRV under controlled resting conditions in healthy subjects [23,24]. The amplitude of the PPG signal is strongly influenced by sympathetically regulated vascular tone in the periphery [40,41]. The PPG-derived estimate of vascular tone is used here as an indirect surrogate of sympathetic influence on the peripheral vasculature, not as a direct measurement of sympathetic nerve activity. Given all of this, we analyzed the influence of SNA and HRV on 0.1 Hz fluctuations through the derived PAC diagrams (Figure 1b and Figure 4). Specifically, we located peaks in 3 min PPG segments using the ‘findpeaks’ function and applied spline interpolation to obtain the RRI curves. Vascular tone was estimated from the peak-to-trough amplitude of each pulse in the PPG signals, suggesting sympathetic nerve activity [41].

We distinguished SNA and HRV in the sense of phase–amplitude coupling by replacing the phase source on the horizontal axis with SNA and RRI signals, obtaining MI matrices of SNA-PPG and RRI-PPG. The 0.1 Hz columns of these MI matrices were extracted for comparison in the range of 0.04–1.4 Hz. The Manhattan distance, correlation distance, and Jensen–Shannon divergence were applied to measure the similarity of these 0.1 Hz couplings. Additional experiments with Euclidean distance yielded the same results as Manhattan; for ease of calculation, the Manhattan distance was retained.

### 2.5. Individual Identification Through MI Matrices Based on Classifiers with the Self-Collected Dataset in the Cross-Session Scenarios

The MI matrices of the self-collected PPG dataset were applied to individual identification to demonstrate the individuality and computational properties of the coupling representation. The PPG waveforms of the left and right fingers often differ morphologically, but their frequency information is essentially the same. Therefore, both channel signals from the same subject were treated as one class. This design encourages the classifier to focus on individual physiological frequency patterns. Considering the relatively small sample size (644 segments), we adopted machine-learning classifiers common in the literature [42] through 6-fold cross-validation, including Support Vector Machine (SVM) [43], K-Nearest Neighbor (KNN) [44], and Convolutional Neural Network (CNN) [45,46]. For SVM and KNN, the two-dimensional MI matrices were flattened into one-dimensional feature vectors as inputs. For a standard 6-fold cross-validation, the inputs (MI matrices for CNN, vectors for SVM and KNN) were partitioned into six folds; each model was trained on five folds and tested on the remaining fold. No additional data reduction or enhancement was performed to determine whether individual PPG signals were sufficiently distinguishable.

For comparison, we additionally employed a template-based method for individual identification from the waveform perspective [42,47,48].

#### 2.5.1. Two-Dimensional CNN Model

**Figure 5:**
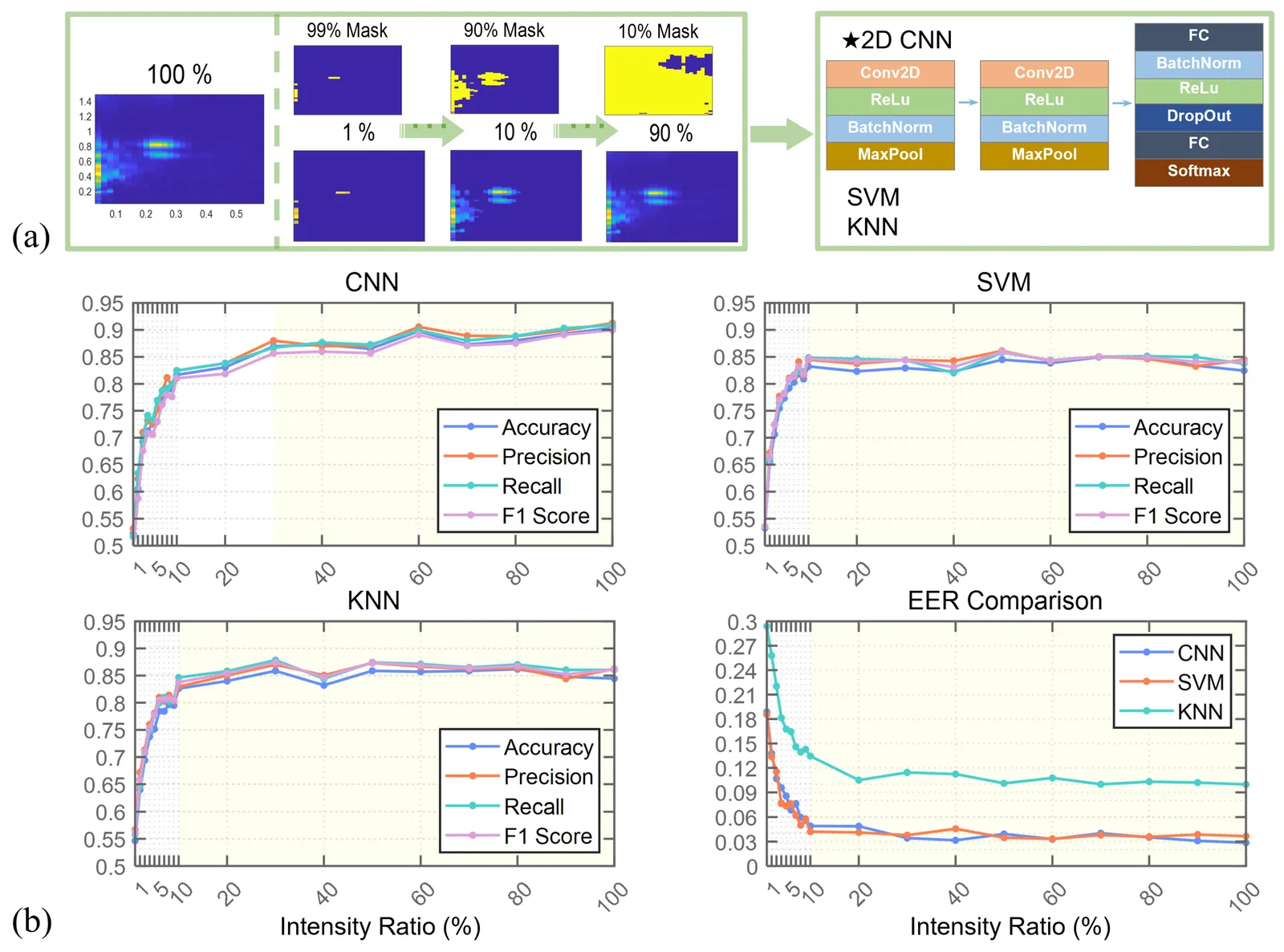
Individual identification performances of different classifiers on MI matrix elements’ intensity retention. (**a**) The MI matrix is thresholded by different ratios of intensity to evaluate the performances of the classifiers (2D CNN, SVM, and KNN), with the remaining elements set to zero. (**b**) Evaluation indicators at different intensity ratios. The classification capability of CNN is close to saturation at ∼30% of matrix elements, while SVM and KNN reach saturation at ∼10%. SVM: Support Vector Machine; KNN: K-Nearest Neighbor; 2D CNN: two-dimensional Convolutional Neural Network.

The MI matrices with structural correlations through the PAC method are well-suited as direct input to a two-dimensional CNN. We used a simple neural network with two layers (Figure 5a). This study focused on the quantitative properties of the coupling matrix from the pulse, rather than on elaborate network construction.

Specifically, each CNN layer contains a sequential two-dimensional convolution operation, a ReLU activation function, a batch normalization layer, and a maximum pooling layer with a pool size of 2 × 2 and a stride of 2. The filter size of the two convolutional layers is 3 × 3, with the ‘Padding’ parameter set to ‘same’, and the number of filters is 32 and 64, respectively. This is followed by a flatten layer and a fully connected layer with 128 outputs. After another batch normalization, ReLU activation, and drop layer with 0.5 probability, there is a fully connected layer with 20 outputs, classified by a softmax layer. In the training process, the Adam optimizer is selected with an initial learning rate of 0.001 and L2 regularization parameter of 0.0001. The maximum number of epochs and batch sizes are 50 and 64, and shuffling is performed at the beginning of each training round.

#### 2.5.2. Template-Based Method

The template-based method requires filtering to reduce noise from baseline shifts or motion artifacts in individual waveforms [42,47,48]. We adopted a zero-phase third-order Butterworth bandpass filter of 0.5–5 Hz. Subsequently, foot-to-foot PPG cycles were separated based on the ‘findpeaks’ function. Each cycle was interpolated using spline interpolation to a width of 125 sampling points, and its amplitude was scaled to the range 0–1 for alignment.

For each 3 min segment, we constructed K (5 or 6) templates based on the number of cycles, ensuring that each template contained 30 complete waveforms. The value of K was determined by numbers of total cycles. The distance between templates measured the similarity of two PPG segments, enabling individual identification. Specifically, the distance between two templates was defined as the minimum Manhattan distance of their stored cycles. The similarity between two signals was described as the minimum value of the distances between their templates. Similarly to the classifier process, we adopted 6-fold cross-validation to construct the repository and performed individual identification. An unregistered signal was classified into the class of the most similar signal stored in the repository.

### 2.6. Individual Identification in the Cross-Day Scenarios

We additionally applied a day-wise (leave-one-day-out) protocol for cross-day evaluation: for each recording day d, each classifier was trained on the sessions of the remaining two days and tested exclusively on the sessions of day d. All classifiers used the same architecture and hyperparameters as in the previous cross-session. The left- and right-hand channels of the same session were treated as one class.

### 2.7. Metrics

The quantitative analysis of frequency components reflects the physiological significance of high-intensity regions in the coupling diagrams. Absolute error was used to describe the difference between frequencies at the maximum MI value and the reference respiration rate and heart rate for each subject from the MARSH dataset. Because the PAC frequency resolution was 0.02 Hz, deviations below 0.02 Hz were considered within the method’s resolution. In addition to mean absolute error, we report linear regression and the Bland–Altman analysis.

For analysis of 0.1 Hz, we applied the Friedman test on the three distance pairs to determine whether similarity differences were significant. Pairwise comparisons were conducted using the Wilcoxon Signed-Rank test with Bonferroni correction.

For individual identification with the self-collected dataset, common evaluation indicators were adopted, including the average accuracy, precision, recall, F1 score, and equal error rate (EER, where false acceptance rate and false rejection rate are equal) of the final model after multiple rounds of training. In addition, to describe the regions with classification significance in PAC diagrams, we retained a certain proportion of MI matrix elements in intensity order, zeroed the rest, and re-evaluated classification performance.

## 3. Results

### 3.1. Digital Pulse Waves Can Be Visually Represented in a Comprehensive Way

We analyzed the PPG signals based on the PAC method to obtain diagrams for holistic visual representation and MI matrices for physiological component extraction and individual identification (Figure 1). High-intensity regions in the diagrams corresponded to clusters of high MI values in the matrices. A close relationship between these regions and physiological frequencies was confirmed, including respiration, heart rate, and 0.1 Hz fluctuations. Individuality in the pulse was demonstrated by machine-learning methods through MI matrices. PAC diagrams divided by different intensity ratios yielded different classification effects and different visually distinguishable patterns.

### 3.2. PAC Diagrams Contain Physiological Information Consistent with Reference Respiration Rate

The physiological information analysis of the PAC diagrams was conducted based on the MARSH dataset. A high-intensity elliptical region in the PAC diagram was observed (Figure 3a), whose horizontal position was consistent with the reference respiratory rate. This region corresponded to the top 5% intensity cluster, with its boundary range indicated by the red band in Figure 3b. The maximum-intensity point in this region is compared with the reference respiration rate (blue point in Figure 3b), and the absolute difference is shown by the solid orange dot at the bottom.

A total of 93.1% (27/29) of reference respiration rates (blue hollow star dots) were included in the significant ellipse ranges. Absolute errors within the PAC calculation step (0.02 Hz) accounted for 75.86% (22/29). With the exception of subject ‘16’ (0.08 Hz difference), the absolute errors of all subjects were below 0.05 Hz. The mean absolute error across the 29 subjects was 0.017 Hz (1.02 breaths/min), with a standard deviation of 0.0158 Hz (0.95 breaths/min).

**Figure 6:**
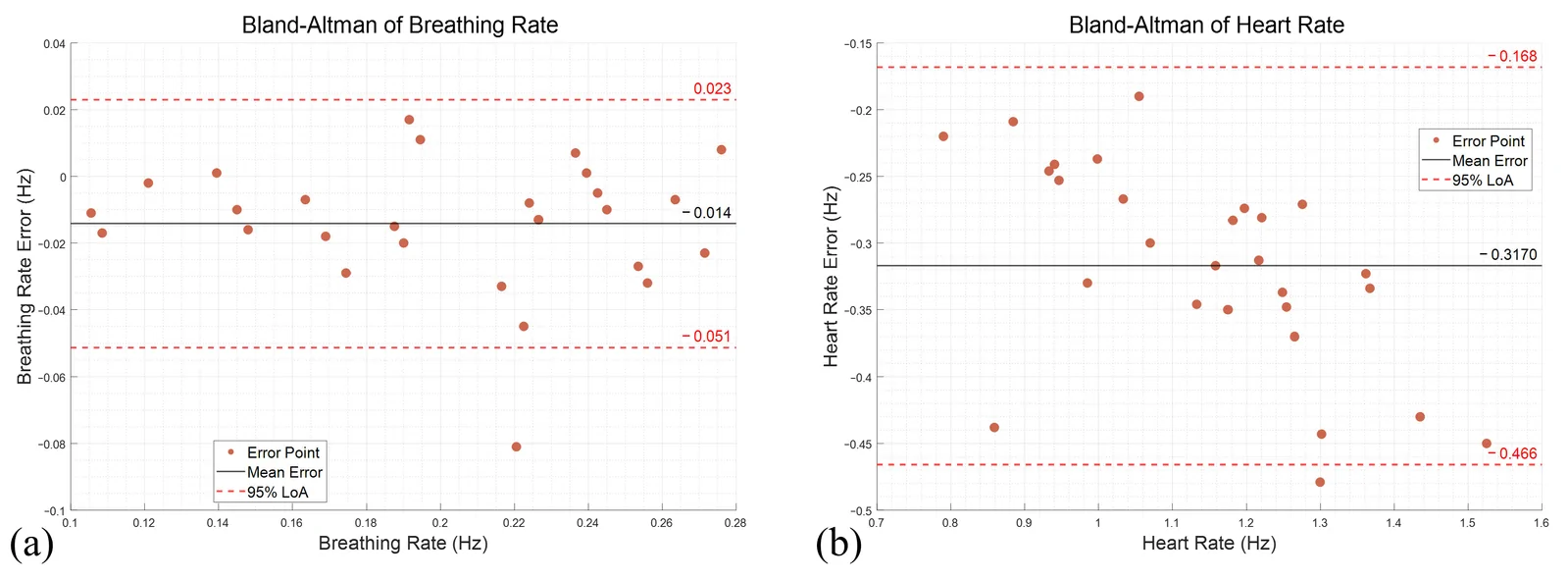
Bland–Altman analysis of breathing rate (**a**) and heart rate (**b**). The gray line marks the mean error (−0.014 Hz for breathing rate and −0.317 Hz for heart rate). Red dashed lines indicate the 95% limits of agreement (LoAs): [−0.051, 0.023] Hz for breathing rate and [−0.466, −0.168] Hz for heart rate.

The R-squared value of the linear regression model is 0.8629. Figure 6a shows the Bland–Altman plot, with 96.6% (28/29) within the 95% limits of agreement. These results confirm that the PAC-derived horizontal frequency is consistent with respiration rate across subjects.

### 3.3. PAC Diagrams Contain Physiological Information Related to Reference Heart Rate

The vertical-axis position in the high-intensity elliptical region was quantitatively compared with the heart rate. Similar to Figure 3b, blue hollow stars and solid points in Figure 3c corresponded to reference heart rates and measured values, respectively. However, a substantial deviation was observed: the absolute frequency differences (orange dots at bottom) had an average of 0.317 Hz (19.02 beats/min) and SD = 0.075 Hz (4.5 beats/min).

Furthermore, the R-squared value of the linear regression model is 0.891. The Bland–Altman plot in Figure 6b shows agreement between PAC and reference measurements, but with broader limits of agreement than that of respiration rate. This relatively constant frequency difference (0.317 Hz) indicates that while the maximum-intensity point may not accurately reflect the true heart rate, it retains a high level of relevant information. The offset likely reflects a harmonic or sub-harmonic interaction in the coupling matrix. The specific mechanism requires further investigation with a simulation experiment and theoretical analysis.

### 3.4. Similarity Analysis at 0.1 Hz in the Sense of Phase–Amplitude Coupling

The 0.1 Hz fluctuation in the coupling diagram was also observed. The right panel of Figure 4a shows PAC pseudo-color diagrams from PPG itself, PPG-derived SNA, and RRI to the PPG signals. All three diagrams contain high-intensity regions related to breathing and heartbeat; however, their intensity distributions differ in the column at 0.1 Hz. Figure 4b presents the numerical distribution of these three columns for 29 subjects in the MARSH dataset, where the horizontal axis is the target amplitude signals coupled at 0.1 Hz with a frequency range of 0.04–1.4 Hz. The strength of PPG self-coupling was lower than the other two at 0.1 Hz for most of the 29 subjects.

Figure 4c further shows intensity differences through three similarity measures, with different subjects on the horizontal axis. In each subgraph, the height of the points in the upper part represents the distance between two pairs of the three columns at 0.1 Hz; higher values indicate lower similarity. Differences between these distances are indicated by the bar charts below. In the left subgraph of Figure 4c, the Manhattan distance gives the sum of point-by-point absolute differences in the curves in Figure 4b. Although the shape and intensity values of the three curves in Figure 4b varied considerably across subjects, from a numerical-distance perspective, SNA and RRI were closer to the PPG signals at 0.1 Hz coupling, as shown by the green dots and light blue bars. The middle subgraph of Figure 4c displays the correlation-distance results, suggesting the degree of linear similarity. At 0.1 Hz, the linear correlation between the coupling curves of SNA and RRI to PPG was the strongest, whereas the coupling of SNA to PPG showed a weaker linear correlation with PPG itself, indicated by green circles. The results of the Jensen–Shannon divergence are shown in the right subgraph of Figure 4c. This measure typically assesses the similarity between two distributions and is less sensitive to ordering than correlation distance. We calculated the Jensen–Shannon divergences after normalizing these curves, and the results also revealed that the coupling distributions at 0.1 Hz from SNA and RRI to PPG were most similar.

Table 1 further shows the p values for similarity differences. The Friedman test was significant (*p* < 0.05) for all three distance measures. For the Wilcoxon Signed-Rank test, with the exception of similarity pairs between (PPG-self vs. SNA-PPG) and (PPG-self vs. RRI-PPG) on Manhattan distance, all similarity differences were significant. This exception corresponds to the left subgraph of Figure 4c.

### 3.5. Individual Identification with MI Matrices

We conducted individual identification based on the self-collected PPG dataset. To analyze the classification significance of regions with different intensities, we reserved a certain proportion of elements in the MI matrix and zeroed the rest. As shown in Figure 5a in the cross-session scenarios, the fixed-parameter classifier was re-evaluated by retaining 1–10% at 1% steps and 10–90% at 10% steps in intensity order, compared with the full (100%) matrix. The changes in five indicators—average accuracy, precision, recall, F1 score and EER—with intensity ratio are displayed in Figure 5b. The fourth subgraph in Figure 5b presents the EER changes in these classifiers. For all indicators, the classification effect of elements within the top 10% of intensity was limited.

For SVM and KNN, classification performance with 10% of MI matrix elements was close to saturation. In Figure 5a, the elements of top 10% intensity visually form significant regions related to respiration rate, heart rate, and 0.1 Hz fluctuations. However, for CNN, the classification effect at a 30% intensity ratio was much closer to saturation than at 10%, which implies that CNN extracts more information from the MI matrix during the classification process than SVM and KNN.

The performances on complete MI matrices are presented in Table 2, along with the template-based method. All values are mean ± standard deviations across six-fold cross-validation.

#### Cross-Day Evaluation

The cross-day evaluations are summarized in Table 3, with all values expressed as mean deviation over the three held-out days. The day-wise accuracies were lower than the session-level ones, but remained markedly above the chance level of 5% for 20 classes.

## 4. Discussion

We achieved a visual representation of digital pulse signals by the PAC method and comprehensively depicted their physiological and individual information. Visual inspection and quantitative validation were performed on physiological frequency components in the coupling diagrams, including breathing rate, heart rate, and 0.1 Hz fluctuations. The results of cross-session individual identification experiments emphasize the computational significance of the modulation index matrices, suggesting that individuality is contained in PPG signals.

PAC diagrams contain holistic information related to various physiological components. In Figure 3b, the significant region associated with breathing rate displayed boundary ranges that varied from person to person. The elliptical morphology may indicate breathing characteristics, such as changing respiration rhythms. Previous studies revealed that different breathing patterns are associated with physical health [49,50] and emotion [51], and they have been validated as potential biometric signatures [43,52]. In addition, there was a deviation of approximately 0.317 Hz between the vertical-axis values and reference heart rates (Figure 3c). This phenomenon was also observed in experiments on other finger PPG datasets, including CapnoBase [34] and BIDMC [53]. Specific reasons require further experiments on real and simulated datasets, as well as a more thorough theoretical understanding of phase–amplitude coupling.

Another significant region is the low-frequency (LF) component, with our focus being at 0.1 Hz, which is confounded by various factors including sympathetic, vagal, and respiratory effects [24,54]. A common view is that regional vasomotion controlled by cutaneous sympathetic oscillations [40,55] dominates the LF component of finger PPG. Analysis in Figure 4c reveals that the effects of SNA and RRI on PPG signals at 0.1 Hz are more similar than other pairs in terms of numerical distance, linear relationship, and distribution. This strongly suggests common influences on SNA and RRI. Studies have shown that breathing has a profound effect on frequency and can overflow to a considerable part of the LF band [24], especially during slow breathing [56,57]. Another result in Figure 4c indicates that the coupling effect between RRI to PPG and PPG itself was less similar than that between SNA and RRI, which may reflect the difference in origin of LF components in finger PPG and HRV [40,55,58]. Kiselev et al. [55] demonstrated that the mechanisms of these two LF oscillations are independent. The coupling from PPG to heart rate may occur through neural pathways, and the reverse coupling implies hemodynamic characteristics. In general, HRV reflects the complex role of cardiac sympathetic and vagus nerve activity [24,59], but great caution is needed when considering the neurogenic factors of the low-frequency component. In a coupling sense, the indirect separation of HRV and SNA from peripheral PPG presented here may supplement and expand current HRV research.

MI matrices exhibit computational attributes for practical applications. The two-dimensional CNN achieved 90.37% accuracy in one-to-many identification across sessions. Most studies perform biometric recognition based on the waveform features of PPG signals [42], which vary considerably in morphology [60]. Different states [48] and cross-session [46] conditions substantially increase the difficulty of the identification task. Table 2 shows that under stable conditions, the template-based method and classifiers performed well; however, the time cost of the template-based method was significantly higher. Compared with the other classifiers in Figure 5b, the KNN method performed worse with a higher EER, possibly because its distance measurement in high-dimensional space required improvement. Frequency-based recognition in this paper is a preliminary exploration, and long-term stability requires continuous experiments to verify the feasibility of longitudinal pulse analysis. In addition, classifiers based on coupling diagrams offer the advantage of physiological interpretability. In Figure 5b, the performances of the classifiers vary with the retention of matrix elements at different intensity ratios. The 10% and 30% retention levels were two near-saturation points. The 10% retention matrix visually covered significant regions related to respiration, heart rate, and 0.1 Hz fluctuations (Figure 5a), indicating that these physiological frequency components may have made considerable contributions to classification. Coupling diagrams inevitably contain visual noise, including motion artifacts; however, some involuntary habits, such as body movement, may be captured by classifiers as additional and potentially beneficial individual characteristics.

However, cross-day identification is considerably more challenging than session-level identification in Table 3, because day-to-day changes in physiological state, sensor placement, and recording conditions may weaken the short-term similarities. This is a recognized challenge in physiological biometric recognition. For PPG-based authentication with a smartphone camera, cross-session EERs of around 20% were reported [61]; ECG-based recognition, although more stable than PPG, still drops from 98–99% (same-session) to 43–56% under cross-session protocols [62]. The cross-day results suggest that the PAC-based MI matrices contain cross-day, subject-specific information, although the absolute performance under the stricter protocol should be interpreted with these cross-session benchmarks in mind.

### Limitations and Future Directions

In addition to the aforementioned discussion about the frequency deviation of physiological components, the in-depth nature of coupling, the mechanism and influence analysis of 0.1 Hz fluctuations, and the extension of individual identification, this study has several other limitations. Firstly, this preliminary study involved limited subjects. The reported accuracy should therefore be interpreted cautiously and not generalized to a larger population. Large-scale studies with hundreds of participants are required to establish the statistical regularities and long-term stability of PPG coupling patterns. Secondly, the 3 min acquisition time may not suit some daily scenarios, such as timely identity authentication. Based on repeated experiments, 3 min is appropriate for conveying visually significant regions, but shorter data lengths may be feasible for classifiers without visual requirements. Another limitation is PPG signal quality. Current calculations are implemented with stable PPG signals on the finger; experiments on wristband datasets with strong noise failed to achieve the expected coupling diagrams for visual representation. In addition, although the classifiers proposed here achieved decent performances, their underlying design requires careful improvement to fully utilize the unique properties of the MI matrices.

Substantial work is required in the future to promote theoretical research and the practical application of PPG signals with the PAC method. We are interested in the relationships between coupling effects conveyed by PAC diagrams and the current concepts of cardiorespiratory coupling, coordination, synchronization, and causation. The effects of respiratory rate, depth, and phase on the phase–amplitude coupling of PPG is another research focus, especially during slow breathing around 0.1 Hz. The difference between state and transition is also a research direction: whether physiological regulation and stress responses can be represented through pulse based on the PAC method. In addition, the temporal stability and continuity of individuality in PPG signals should be determined [16], exploiting the long-term tracking advantages of wearable technology, in both cross-session and cross-day scenarios. This could facilitate further longitudinal trajectory analysis [13] and early warning and intervention for adverse physiological events [11,16,17]. We will explore the combination of routine data with regular clinical measurements [10,63] to provide more reliable medical interpretations [5] and facilitate interdisciplinary collaboration. Multimodal signals [9,10,13], integrating PPG with other wearables, also provide opportunities for multi-dimensional health maintenance. We expect to converge advanced technologies, such as machine learning [12], to improve the evaluation of physiological signals and the analysis of digital health. Moreover, large-scale population studies would help elucidate the statistical regularities of PPG coupling with physical health and mental states.

## 5. Conclusions

In this paper, we presented a preliminary exploration of physiological information and individuality from wearable PPG signals using phase–amplitude coupling representation. By applying the PAC method, we provided an overall description of relevant physiological components of PPG signals—including respiration rate, heart rate, and 0.1 Hz fluctuations—which may enhance the understanding of HRV. The cross-session individual identification demonstrated the discriminative power of the modulation index matrices. When combined with routine clinical measurements, integrated with multiple devices, and fused with machine-learning technologies, this comprehensive analysis provides conceptual and methodological foundations for realizing personalized health.

## Figures and Tables

**Table 1:** *p* values of Friedman test and Wilcoxon Signed-Rank test for three similarity measures.

Similarity Measures	Friedman Test	Wilcoxon Signed-Rank Test ^1^		
		Dist1 vs. Dist2	Dist1 vs. Dist3	Dist3 vs. Dist2
Manhattan Distance	2.3579 × 10^−5^	3.5810 × 10^−1 2^	1.2398 × 10^−4^	4.4179 × 10^−4^
Correlation Distance	6.2618 × 10^−8^	1.8970 × 10^−2^	4.3289 × 10^−6^	2.8631 × 10^−5^
Jensen–Shannon Divergence	6.2618 × 10^−8^	9.1715 × 10^−3^	4.8009 × 10^−6^	1.7691 × 10^−5^

^1^ Dist1, Dist2, and Dist3 represent the coupling similarities of PPG-self vs. SNA-PPG, PPG-self vs. RRI-PPG, and SNA-PPG vs. RRI-PPG. ^2^ >0.05.

**Table 2:** Performances of different classifiers and template-based methods on individual identification (mean ± SD) in the cross-session scenarios. SVM: Support Vector Machine; KNN: K-Nearest Neighbor; 2D CNN: two-dimensional Convolutional Neural Network.

Indicators (%)	Accuracy	Precision	Recall	F1 Score	EER
CNN	90.37 ± 3.60	91.25 ± 2.46	90.86 ± 3.61	89.95 ± 3.10	2.86 ± 1.12
SVM	82.44 ± 3.50	84.47 ± 3.41	83.74 ± 3.32	84.08 ± 3.26	3.66 ± 1.35
KNN	84.45 ± 4.15	86.25 ± 3.14	86.06 ± 3.82	86.15 ± 3.43	9.97 ± 2.01
Template-based	85.86 ± 2.36	86.26 ± 3.28	85.54 ± 2.71	85.89 ± 2.98	-

**Table 3:** Performances of different classifiers on individual identification (mean) in the cross-day scenarios. SVM: Support Vector Machine; KNN: K-Nearest Neighbor; 2D CNN: two-dimensional Convolutional Neural Network.

Indicators (%)	Accuracy	Precision	Recall	F1 Score	EER
CNN	56.32	57.66	56.72	57.18	14.39
SVM	52.04	52.82	52.31	52.55	19.14
KNN	46.25	48.45	46.31	47.26	28.26

## Data Availability

The MARSH dataset [35] is available at https://zenodo.org/records/3673924 (accessed on 20 August 2026). The self-collected datasets are available on reasonable request from the corresponding author.

## Abbreviations

The following abbreviations are used in this manuscript:

PPG	Photoplethysmography
PAC	Phase–amplitude coupling
MI	Modulation index
DPPG	Differential photoplethysmography
MAX MI	Maximum modulation index values
Moran’s I	Moran index
HRV	Heart rate variability
CDF	Cumulative distribution function
SVM	Support Vector Machine
KNN	K-Nearest Neighbor
CNN	Convolutional Neural Network
EER	Equal error rate
SNA	Sympathetic nerve activity
RRI	R-R interval
LF	Low-frequency

## References

[1] Konstantinidou M., Karaglani M., Panagopoulou M., Fiska A., Chatzaki E. (2017). Are the Origins of Precision Medicine Found in the Corpus Hippocraticum?. Mol. Diagn. Ther..

[2] Visvikis-Siest S., Theodoridou D., Kontoe M.S., Kumar S., Marschler M. (2020). Milestones in Personalized Medicine: From the Ancient Time to Nowadays—The Provocation of COVID-19. Front. Genet..

[3] Collins F., Varmus H. (2015). A New Initiative on Precision Medicine. N. Engl. J. Med..

[4] Jameson J., Longo D. (2015). Precision Medicine—Personalized, Problematic, and Promising. N. Engl. J. Med..

[5] Foy B., Petherbridge R., Roth M., Zhang C., De Souza D., Mow C., Patel H., Patel C., Ho S., Lam E. (2024). Haematological setpoints are a stable and patient-specific deep phenotype. Nature.

[6] Spear B., Heath-Chiozzi M., Huff J. (2001). Clinical Applications of Pharmacogenetics. Trends Mol. Med..

[7] Schork N. (2015). Personalized medicine: Time for one-person trials. Nature.

[8] Kaaronen R., Manninen M.A., Eronen J. (2024). Measuring Beyond the Standard: Informal Measurement Systems as Cognitive Technologies. Top. Cogn. Sci..

[9] Gambhir S., Ge T., Vermesh O., Spitler R. (2018). Toward achieving precision health. Sci. Transl. Med..

[10] Yurkovich J., Evans S., Rappaport N., Boore J., Lovejoy J., Price N., Hood L. (2023). The transition from genomics to phenomics in personalized population health. Nat. Rev. Genet..

[11] Phipps J., Passage B., Sel K., Martínez J., Saadat M., Koker T., Damaso N., Davis S., Palmer J., Claypool K. (2024). Early adverse physiological event detection using commercial wearables: Challenges and opportunities. npj Digit. Med..

[12] Sameh A., Rostami M., Oussalah M., Korpelainen R., Farrahi V. (2024). Digital phenotypes and digital biomarkers for health and diseases: A systematic review of machine learning approaches utilizing passive non-invasive signals collected via wearable devices and smartphones. Artif. Intell. Rev..

[13] Li X., Dunn J., Salins D., Zhou G., Zhou W., Schüssler-Fiorenza Rose S.M., Perelman D., Colbert E., Runge R., Rego S. (2017). Digital Health: Tracking Physiomes and Activity Using Wearable Biosensors Reveals Useful Health-Related Information. PLoS Biol..

[14] Dunn J., Kidzinski L., Runge R., Witt D., Schüssler-Fiorenza Rose S.M., Bahmani A., Delp S., Hastie T., Snyder M. (2021). Wearable sensors enable personalized predictions of clinical laboratory measurements. Nat. Med..

[15] Walter J., Xu S., Rogers J. (2024). From lab to life: How wearable devices can improve health equity. Nat. Commun..

[16] Schüssler-Fiorenza Rose S.M., Contrepois K., Moneghetti K., Zhou W., Mishra T., Mataraso S., Dagan-Rosenfeld O., Ganz A., Dunn J., Hornburg D. (2019). A longitudinal big data approach for precision health. Nat. Med..

[17] Alavi A., Bogu G., Wang M., Rangan E., Brooks A., Wang Q., Higgs E., Celli A., Mishra T., Metwally A. (2022). Real-time alerting system for COVID-19 and other stress events using wearable data. Nat. Med..

[18] Mahato K., Saha T., Ding S., Sandhu S., Chang A.Y., Wang J. (2024). Hybrid multimodal wearable sensors for comprehensive health monitoring. Nat. Electron..

[19] Celik Y., Godfrey A. (2023). Bringing it all together: Wearable data fusion. npj Digit. Med..

[20] Allen J. (2007). Photoplethysmography and its application in clinical physiological measurement. Physiol. Meas..

[21] Charlton P., Birrenkott D., Bonnici T., Pimentel M., Johnson A., Alastruey J., Tarassenko L., Watkinson P., Beale R., Clifton D. (2017). Breathing Rate Estimation From the Electrocardiogram and Photoplethysmogram: A Review. IEEE Rev. Biomed. Eng..

[22] Khoo M., Chalacheva P. (2019). Respiratory modulation of peripheral vasoconstriction: A modeling perspective. J. Appl. Physiol..

[23] Mejía-Mejía E., May J., Torres R. (2020). Pulse rate variability in cardiovascular health: A review on its applications and relationship with heart rate variability. Physiol. Meas..

[24] Quigley K., Gianaros P., Norman G., Jennings J., Berntson G., Geus E. (2024). Publication guidelines for human heart rate and heart rate variability studies in psychophysiology-Part 1: Physiological underpinnings and foundations of measurement. Psychophysiology.

[25] Wang W., Mohseni P., Kilgore K.L., Najafizadeh L. (2022). Cuff-Less Blood Pressure Estimation From Photoplethysmography via Visibility Graph and Transfer Learning. IEEE J. Biomed. Health Inform..

[26] Lacasa L., Luque B., Ballesteros F., Luque J., Nuno J.C. (2008). From time series to complex networks: The visibility graph. Proc. Natl. Acad. Sci. USA.

[27] Tiwari A., Gray G., Bondi P., Mahnam A., Falk T.H. (2023). Automated Multi-Wavelength Quality Assessment of Photoplethysmography Signals Using Modulation Spectrum Shape Features. Sensors.

[28] Tort A.B.L., Komorowski R., Eichenbaum H., Kopell N. (2010). Measuring Phase-Amplitude Coupling Between Neuronal Oscillations of Different Frequencies. J. Neurophysiol..

[29] Onslow A., Bogacz R., Jones M. (2011). Quantifying phase—Amplitude coupling in neuronal network oscillations. Prog. Biophys. Mol. Biol..

[30] Hülsemann M., Naumann E., Rasch B. (2019). Quantification of Phase-Amplitude Coupling in Neuronal Oscillations: Comparison of Phase-Locking Value, Mean Vector Length, Modulation Index, and Generalized-Linear-Modeling-Cross-Frequency-Coupling. Front. Neurosci..

[31] Richter C., Babo-Rebelo M., Schwartz D., Tallon-Baudry C. (2016). Phase-amplitude coupling at the organism level: The amplitude of spontaneous alpha rhythm fluctuations varies with the phase of the infra-slow gastric basal rhythm. NeuroImage.

[32] Kluger D., Gross J. (2021). Respiration modulates oscillatory neural network activity at rest. PLoS Biol..

[33] Sargent K., Martinez E., Reed A., Guha A., Bartholomew M., Diehl C., Chang C., Salama S., Popov T., Thayer J. (2024). Oscillatory Coupling Between Neural and Cardiac Rhythms. Psychol. Sci..

[34] Karlen W., Raman S., Ansermino M., Dumont G. (2013). Multiparameter Respiratory Rate Estimation From the Photoplethysmogram. IEEE Trans. Bio-Med. Eng..

[35] Pirhonen M., Vehkaoja A. (2020). Fusion enhancement for tracking of respiratory rate through intrinsic mode functions in photoplethysmography. Biomed. Signal Process. Control.

[36] Moran P.A.P. (1950). Notes on Continuous Stochastic Phenomena. Biometrika.

[37] Mohamed E. (2016). Optimal Signal Quality Index for Photoplethysmogram Signals. Bioengineering.

[38] Liang Y., Elgendi M., Chen Z., Ward R. (2018). An optimal filter for short photoplethysmogram signals. Sci. Data.

[39] Shaffer F., Ginsberg J. (2017). An Overview of Heart Rate Variability Metrics and Norms. Front. Public Health.

[40] Chan G., Fazalbhoy A., Birznieks I., Macefield V., Middleton P., Lovell N. (2011). Spontaneous fluctuations in the peripheral photoplethysmographic waveform: Roles of arterial pressure and muscle sympathetic nerve activity. Am. J. Physiol. Heart Circ. Physiol..

[41] Özbay P., Chang C., Picchioni D., Mandelkow H., Chappel-Farley M., Gelderen P., de Zwart J., Duyn J. (2019). Sympathetic activity contributes to the fMRI signal. Commun. Biol..

[42] Labati R., Piuri V., Rundo F., Scotti F. (2022). Photoplethysmographic Biometrics: A Comprehensive Survey. Pattern Recognit. Lett..

[43] Hinatsu S., Suzuki D., Ishizuka H., Ikeda S., Oshiro O. Photoplethysmographic Subject Identification by Considering Feature Values Derived from Heartbeat and Respiration. Proceedings of the 2020 42nd Annual International Conference of the IEEE Engineering in Medicine & Biology Society (EMBC).

[44] Jaafar N.A.L., Sidek K.A., Azam S.N.A.M. Acceleration plethysmogram based biometric identification. Proceedings of the International Conference on BioSignal Analysis, Processing and Systems (ICBAPS).

[45] Biswas D., Everson L., Liu M., Panwar M., Verhoef B.E., Patrika S., Kim C., Acharyya A., Van Hoof C., Konijnenburg M. (2019). CorNET: Deep Learning Framework for PPG-Based Heart Rate Estimation and Biometric Identification in Ambulant Environment. IEEE Trans. Biomed. Circuits Syst..

[46] Hwang D.Y., Taha B., Lee D.S., Hatzinakos D. (2021). Evaluation of the Time Stability and Uniqueness in PPG-Based Biometric System. IEEE Trans. Inf. Forensics Secur..

[47] Jorge S., álvaro A., José G. (2018). Biometric Authentication Using the PPG: A Long-Term Feasibility Study. Sensors.

[48] Yadav U., Abbas S.N., Hatzinakos D. Evaluation of PPG Biometrics for Authentication in Different States. Proceedings of the 2018 International Conference on Biometrics (ICB).

[49] Liang W., Ji Y.X., Xiao J., Truskauskaitė I., Hendrixson A., Bai Z.M., Ruksenas O. (2024). Respiratory patterns and physical fitness in healthy adults: A cross-sectional study. BMC Public Health.

[50] Gorodisky L., Honigstein D., Weissbrod A., Weissgross R., Soroka T., Shushan S., Sobel N. (2024). Humans without a sense of smell breathe differently. Nat. Commun..

[51] Oku Y. (2022). Temporal variations in the pattern of breathing: Techniques, sources, and applications to translational sciences. J. Physiol. Sci..

[52] Jagmohan C., Hu Y., Seneviratne S., Misra A., Seneviratne A., Lee Y. BreathPrint: Breathing Acoustics-based User Authentication. Proceedings of the 15th Annual International Conference on Mobile Systems, Applications, and Services.

[53] Pimentel M., Johnson A., Charlton P., Birrenkott D., Watkinson P., Tarassenko L., Clifton D. (2016). Toward a Robust Estimation of Respiratory Rate From Pulse Oximeters. IEEE Trans. Biomed. Eng..

[54] Ackermann S., Laborde S., Borges U., Mosley E. (2021). Commentary: Photoplethysmography for Quantitative Assessment of Sympathetic Nerve Activity (SNA) During Cold Stress. Front. Physiol..

[55] Kiselev A., Borovkova E., Shvartz V., Skazkina V., Karavaev A., Prokhorov M., Ispiryan A., Mironov S., Bockeria O. (2020). Low-frequency variability in photoplethysmographic waveform and heart rate during on-pump cardiac surgery with or without cardioplegia. Sci. Rep..

[56] Stankovski T., Cooke W., Rudas L., Stefanovska A., Eckberg D. (2013). Time-frequency methods and voluntary ramped-frequency breathing: A powerful combination for exploration of human neurophysiological mechanisms. J. Appl. Physiol..

[57] Tankanag A., Krasnikov G., Mizeva I. (2020). A pilot study: Wavelet cross-correlation of cardiovascular oscillations under controlled respiration in humans. Microvasc. Res..

[58] Karavaev A., Borovik A., Borovkova E., Orlova E., Simonyan M., Ponomarenko V., Skazkina V., Gridnev V., Bezruchko B., Prokhorov M. (2021). Low-frequency component of photoplethysmogram reflects the autonomic control of blood pressure. Biophys. J..

[59] Laborde S., Mosley E., Thayer J. (2017). Heart Rate Variability and Cardiac Vagal Tone in Psychophysiological Research—Recommendations for Experiment Planning, Data Analysis, and Data Reporting. Front. Psychol..

[60] Hickey M., Phillips J. (2015). Investigation of peripheral photoplethysmographic morphology changes induced during a hand-elevation study. J. Clin. Monit. Comput..

[61] Lovisotto G., Turner H., Eberz S., Martinovic I. Seeing Red: PPG Biometrics Using Smartphone Cameras. Proceedings of the 2020 IEEE/CVF Conference on Computer Vision and Pattern Recognition Workshops (CVPRW).

[62] Islameddine A., Amir B., Abdelhafid Z., Lotfi H. (2026). Attention-guided fusion of 1D and 2D CNNs for robust ECG-based biometric recognition. Digit. Signal Process..

[63] Friligkou E., Koller D., Pathak G., Miller E., Lampert R., Stein M., Polimanti R. (2025). Integrating genome-wide information and wearable device data to explore the link of anxiety and antidepressants with pulse rate variability. Mol. Psychiatry.

